# Draft Genome Sequence of Corynebacterium aurimucosum UMB7769, Isolated from the Female Urinary Tract

**DOI:** 10.1128/MRA.00391-20

**Published:** 2020-05-28

**Authors:** Samantha Eskandar, Taylor Miller-Ensminger, Adelina Voukadinova, Alan J. Wolfe, Catherine Putonti

**Affiliations:** a Department of Biology, Loyola University Chicago, Chicago, Illinois, USA; b Bioinformatics Program, Loyola University Chicago, Chicago, Illinois, USA; c Department of Microbiology and Immunology, Stritch School of Medicine, Loyola University Chicago, Maywood, Illinois, USA; d Department of Computer Science, Loyola University Chicago, Chicago, Illinois, USA; University of Maryland School of Medicine

## Abstract

Here, we present the draft genome sequence of Corynebacterium aurimucosum UMB7769, isolated from the female urinary tract. The size of the genome is 2,731,818 bp, assembled in 50 contigs, with an observed GC content of 60.9% and an *N*_50_ score of 129,518 bp. Annotation revealed 31 antibiotic resistance genes.

## ANNOUNCEMENT

The prevalence of *Corynebacterium* in urine has been noted in both healthy and symptomatic male and female patients (1–4). Relative to other bacteria in the urobiome, relatively little is known about C. aurimucosum. Recently, the presence of *C. aurimucosum* in urine was associated with urinary tract infections (UTI) (5). A subsequent case study explored the possible infectious role of *C. aurimucosum* due to its presence in a patient with a UTI (6). Here, we report the genome sequence of *C. aurimucosum* UMB7769, isolated from the voided urine of a woman with a recurrent UTI.

*C. aurimucosum* UMB7769 was isolated from a prior institutional review board (IRB)-approved study (University of California, San Diego, IRB no. 170077AW) using the expanded quantitative urinary culture (EQUC) protocol (1). The sample was collected from a patient at the Women’s Pelvic Medicine Center at the University of California, San Diego, in August 2017. One hundred microliters of urine was spread onto a 5% sheep blood agar plate (BAP) and incubated at 35°C in a 5% CO_2_ environment for 24 h. Each distinct colony morphology on this plate was subcultured to obtain a pure culture for microbial identification. Matrix-assisted laser desorption ionization–time of flight (MALDI-TOF) mass spectrometry was used to determine the genus and species of this isolate, following protocols detailed previously (1). The isolate was then stored at −80°C until sequencing. From this freezer stock, the *C. aurimucosum* isolate was streaked onto a Columbia nalidixic acid (CNA) agar plate using the four-quadrant streaking method and incubated at 35°C with 5% CO_2_ for 24 h. A single colony was selected and grown in LB overnight under the same conditions. DNA was extracted from this liquid culture using the Qiagen DNeasy blood and tissue kit with a few modifications to the Gram-positive bacterium protocol: in step 2, 230 μl of lysis buffer (180 μl of 20 mM Tris-Cl, 2 mM sodium EDTA, and 1.2% Triton X-100 and 50 μl of lysozyme) was used to resuspend the pellet. The incubation time in step 5 was also shortened from 30 to 10 min. Purified DNA was quantified using a Qubit fluorometer. DNA was sent to the Microbial Genome Sequencing Center (MiGS) at the University of Pittsburgh for sequencing, where the DNA was first enzymatically fragmented using an Illumina tagmentation enzyme. Indices were attached using PCR and then sequenced using an Illumina NextSeq 550 flow cell. In total, 1,041,570 pairs of 150-bp reads were produced for the strain. The raw reads were trimmed using Sickle v1.33 (https://github.com/najoshi/sickle). The reads were assembled using SPAdes v3.13.0 with the “only-assembler” option for k values of 55, 77, 99, and 127 (7). The genome was annotated using PATRIC v3.6.3 (8); however, the publicly available genome was annotated using Prokaryotic Genome Annotation Pipeline (PGAP) v4.11 (9). Unless previously noted, default parameters were used for each software tool.

The *C. aurimucosum* UMB7769 draft genome is 2,731,818 bp, assembled in 50 contigs, with a GC content of 60.9%. The assembly has a genome coverage of 99× and an *N*_50_ score of 129,518 bp. PATRIC annotation identified 31 antibiotic resistance genes, one of which encodes resistance to tetracycline. This resistance was also confirmed by ResFinder v3.2 (10). The PGAP annotation also identified 2,486 protein-coding genes, 52 tRNAs, and 4 rRNA operons. Through further isolation, study, and sequencing of *C. aurimucosum* strains from the urinary tract, we hope to ascertain whether this species should be considered an emerging pathogen.

### Data availability.

This whole-genome shotgun project has been deposited in GenBank under the accession no. JAAUWA000000000. The version described in this paper is the first version, JAAUWA010000000. The raw sequencing reads have been deposited in the SRA under the accession no. SRR11441031.
